# Emergency Fostering of Dogs From Animal Shelters During the COVID-19 Pandemic: Shelter Practices, Foster Caregiver Engagement, and Dog Outcomes

**DOI:** 10.3389/fvets.2022.862590

**Published:** 2022-04-27

**Authors:** Lisa M. Gunter, Rachel J. Gilchrist, Emily M. Blade, Jenifer L. Reed, Lindsay T. Isernia, Rebecca T. Barber, Amanda M. Foster, Erica N. Feuerbacher, Clive D. L. Wynne

**Affiliations:** ^1^Department of Psychology, Arizona State University, Tempe, AZ, United States; ^2^Department of Animal and Poultry Sciences, Virginia Polytechnic Institute and State University (Virginia Tech), Blacksburg, VA, United States; ^3^Division of Education Leadership and Innovation, Arizona State University, Tempe, AZ, United States

**Keywords:** dog, animal shelter, foster care, COVID-19 pandemic, welfare, adoption, emergency

## Abstract

Each year, millions of dogs enter thousands of animal shelters across the United States. Life in the shelter can be stressful, and one type of intervention that improves dogs' experience is human interaction, particularly stays in foster homes. Prior research has demonstrated that fostering can reduce dogs' cortisol and increase their resting activity. Despite these benefits, little is understood about the utilization of foster caregiving in animal shelters, and even less so during a crisis. On March 11, 2020, the World Health Organization deemed the coronavirus outbreak a worldwide pandemic, and subsequently a nationwide emergency was declared in the United States. Nearly all states issued stay-at-home orders to curb the spread of the virus. During this time, media outlets reported increased interest in the adoption and fostering of shelter pets. This study explores canine foster caregiving at 19 US animal shelters during the first 4 months of the COVID-19 pandemic. In our investigation, we found that shelters' utilization of foster caregiving increased from March to April 2020 but returned to initial pandemic levels by June 2020. Slightly less than two-fifths of foster caregivers were community members with no prior relationship with the shelter, and these caregivers were over four times more likely to adopt their fostered dogs than those with a pre-existing relationship to the shelter. Individuals fostering with the intention to adopt, in fact, adopted their dogs in nearly three-quarters of those instances. With regards to shelters' available resources, we found that very low-resource shelters relied more heavily on individuals with prior relationships to provide foster caregiving while very high-resource shelters more often recruited new community members. We also found that our lowest resourced shelters transferred more dogs out of their facilities while more resourced shelters rehomed dogs directly to adopters. To our knowledge, these findings represent the first in-depth reporting about dog fostering in US animal shelters and, more specifically, foster caregiving during the COVID-19 pandemic. In total, they provide greater understanding of how monetary and human resources were utilized to affect the care and ultimately, the outcomes of shelter dogs during this time.

## Introduction

Each year, millions of dogs enter thousands of animal shelters across the United States. These dogs are most often rehomed to new adopters with a smaller proportion returned to their owners, and yet a smaller proportion euthanized (1–3). While in the animal shelter, dogs' daily experience is stressful when compared to that of dogs living in homes (4, 5), likely due to the excessive noise in kenneling areas, restrictions to movement, loss of control, lack of a routine, and social isolation [for more about these issues, see (6)]. Cortisol levels for dogs living in shelters are elevated, and dogs are less rested in this environment than homes (6–8).

Enrichment interventions, aimed at improving the dogs' proximate welfare, have been successful in improving this daily experience (9–11). Human interactions are one of the most well-studied and consistently effective interventions in the animal shelter [see (12) for a review]. One type of human-interaction intervention, foster caregiving, allows dogs to leave the shelter for a period of time, escaping the stressors of this environment. Gunter et al. (6) found that one- and two-night stays with foster caregivers resulted in cortisol reductions while in the home; and although dogs' cortisol did rise upon return to the shelter, those levels were no higher than their baseline values prior to fostering. Fehringer (13) also demonstrated that 3 days in a foster caregivers' home lowered dogs' cortisol compared to levels measured in the shelter. However, it appears that brief excursions from the animal shelter do not confer the same benefits, as dogs provided only two-and-a-half hour outings away from the shelter (typically into the community but not in a home) led to higher cortisol, not lower, even after accounting for the dogs' overall activity levels (12).

Time in a home is likely beneficial for dogs' proximate welfare as they await adoption, but it is also possible that fostering affects their ultimate welfare by facilitating placement into an adoptive home (14, 15). Trial adoption programs, in which dogs are cared for by individuals who are interested in adopting them, have been shown to reduce the likelihood of return by adopters (14). Return rates are also lower when dogs are fostered by caregivers who are responsible for their placement. Mohan-Gibbons and colleagues found that adopters of fostered dogs used information provided by foster caregivers in their adoption decision-making more often than adopters of shelter dogs used information provided by the staff who were caring for the dogs. They also found that the adopters of fostered dogs resided in different areas of the community than those adopting directly from the shelter, suggesting that placing animals in the homes of foster caregivers can expand the visibility of animals awaiting adoption (15).

Despite the potential benefits of foster caregiving, little has been characterized in the scientific literature about the prevalence and utilization of these programs in animal sheltering. In a recent survey of US animal shelters and rescues, fewer than half of the responding organizations had foster programs for their homeless pets and placed very few dogs in foster care (16). In a study of a municipal animal shelter in the American Southwest, Patronek and Crowe (17) reported that <10% of dogs that entered the shelter from 2015 to 2016 were placed in foster care, with many of the dogs needing behavioral or medical treatment prior to adoption. Nearly 98% of these fostered dogs had a live outcome (i.e., adoption or transfer to another agency) compared with the overall rate for dogs that was under 90%.

Over 90 million households in the US, equaling 70% of all households in the country, own a pet (18). Hazardous events, such as natural disasters, can have profound effects on the lives of pet owners and their animals. During Hurricane Andrew in 1992, tens of thousands of pets were abandoned in southeast Florida, and over a thousand dogs and cats were euthanized because animal welfare agencies had nowhere to house them (19). Since the 1990s, animal sheltering's response to natural disasters has improved and continues to do so. For example, when Hurricane Charley impacted southwest Florida in 2004, the euthanasia of pets because of a lack of physical space was virtually non-existent, due in part to a coordinated emergency response and a local network of foster caregivers who took animals into their homes (20). Hurricane Katrina and its aftermath impacted hundreds of thousands of owners and their pets, but the event was a catalyst for change. Emergency management and disaster response became more inclusive of people with their pets. Animal transport, born out of the necessity of moving dogs and cats out of hurricane-affected areas, has now grown into a vast network of animal shelters and rescues, moving animals from in-need shelters to those that are more resourced (21, 22).

On March 11, 2020, the World Health Organization (WHO) declared the coronavirus outbreak a worldwide pandemic (23); and 2 days later, the US president declared a nationwide emergency with all states approved for disaster assistance (24). Unlike other types of disasters, the COVID-19 pandemic differed in significant ways for people and their pets. It was not a localized geographic or meteorological event, and there was no widespread destruction of infrastructure. Instead, animal shelters, pet owners, and foster caregivers continued to have the capacity to house their pets. In March and April 2020, 90% of American states or parts of American states issued stay-at-home orders for their residents (25). This resulted in an unprecedented number of people remaining in their domiciles, discouraged to travel or physically interact with non-cohabiting friends and family.

During this initial phase of the pandemic, veterinary medicine programs specializing in the care of animals in shelters recommended decreasing the number of on-site staff to help curb the spread of the disease. To do this, shelters were encouraged to reduce the intake of new animals, find adoptive homes for the animals that were living in the shelter, and place the remaining pets into foster care (26). As such, many shelters implemented these operational changes, including seeking new foster caregivers within their communities (27). Media outlets reported that animal shelters across the United States were receiving increased inquiries about fostering and the adoption of pets (28, 29). In the present study, we explored the utilization of foster caregiving at 19 US animal shelters during the first 4 months of the COVID-19 pandemic. Additionally, we examined qualities of the shelters and foster caregivers that were related to foster care utilization as well as the outcomes for dogs that participated in these foster programs.

## Materials and Methods

### Animal Shelters

Animal shelters utilizing canine fostering in March 2020 were eligible to participate in the study. Organizations and their staff that expressed interest in participation, in response to email and online announcements, were contacted, and those that were able to collect data about their canine foster care programs were enrolled in the study.

These shelters provided demographic information about their organizations, including location, admission policy (i.e., open, managed, or limited admission), organization type (i.e., municipal, private non-profit, or private non-profit with municipal contracts), and 2020 operating budget. Open admission was defined as those facilities with unrestricted intake of animals in the areas they served. Shelters with managed admission policies controlled the arrival of animals coming into their facilities while limited admission shelters restricted the animals accepted into care (30). We also collected the number of animals that were brought into the shelters in 2019 as well as that year's live release rate for dogs (calculated by dividing the total live outcomes for dogs by all outcomes for dogs that came into care).

To learn more about the shelter's processes and procedures, we queried the shelters about the presence of a foster program prior to the pandemic, whether they conducted behavioral and dog-to-dog assessments, as well as whether the organization had behavior staff. We also asked whether the shelter had reduced the number of in-house veterinarians able to perform spay-neuter surgeries during the first 4 months of the COVID-19 pandemic and whether such surgeries were required prior to adoption. Additionally, we gathered more information about adoption procedures during this time, including their requirements for meeting adoptable dogs, the handling of adoption paperwork, and how the physical acquisition of the animal was conducted. (Adoption procedures about paperwork handling and animal acquisition methods were not mutually exclusive categorical variables).

### Dogs and Foster Caregivers

Dogs placed into foster care and included in this study's dataset did so from the date of the WHO's declaration of a global COVID-19 pandemic, March 11, 2020, until June 30, 2020. Animal shelter staff determined which dogs in their care were suitable for fostering, identified dogs with continuing behavioral or medical needs as well as the criteria and training for foster caregivers. While each shelter determined what defined ongoing needs, common behavioral issues included dogs that were shy or nervous around people, did not get along with other dogs, or were failing to thrive in the shelter environment. Continuing medical needs often included dogs that were on medication, such as antibiotics, treatment for ear or eye infections, or pain control, or monitoring post surgery. The duration of foster care was decided upon by shelter staff and their caregivers.

Foster experiences were categorized as being either puppy fostering (dogs under 8 weeks of age) or dog fostering (dogs 8 weeks and older). Data about dogs that were fostered by potential adopters (people primarily interested in adopting those dogs) were also collected, though on a more limited basis, as these experiences were typically carried out by adoption, not foster, department staff.

We collected information about the animals using the shelter's database system and other sources including intake date and type (i.e., stray, owner surrender, returned adoption, transfer in), estimated date of birth, sex, weight, date of spay-neuter (if not already altered when brought into the shelter), and the animal's outcome (i.e., adoption, rescue/transfer, euthanasia). For dogs that were adopted by their foster caregivers, length of stay calculations ended when they notified staff of their intention to adopt, in order to account for additional time processing paperwork or other shelter procedures that may increase these lengths of stay.

Additionally, we categorized the timing of an animal's spay-neuter surgery (based on intake, surgery, foster entrance and exit dates) as having occurred: (1) prior to arriving at the shelter, (2) in the shelter, (3) during foster care, (4) while in a foster-to-adopt, (5) after a foster caregiver informed the shelter of their intention to adopt, or (6) after the dog was adopted or transferred to a rescue. Shelter staff also indicated whether the animal bit a person or dog during its foster stay and the reason foster care ended, such as adoption, a scheduled return as coordinated by the shelter, or a behavioral, medical, or caregiver-related issue. Table 1 describes these categorizations and specified reasons associated with them.

**Table 1:** Categorization of reasons for the return of a fostered dog.

**Category**	**Specific reasons**
Adoption	Fostered dog is being adopted
Behavioral	Behavior of the fostered dog and/or resident pet(s) has become undesirable or unmanageable during fostering
Medical	Fostered dog has undesirable or unmanageable medical needs
Caregiver-related	Travel, change in schedule, change in housing, personal circumstances of foster caregiver, foster experience was not meeting caregiver's expectations, health of the foster caregiver, their household, or resident pet(s)
Scheduled return by the shelter	Adoption marketing of fostered dog, transfer to another animal welfare organization, placement with another foster caregiver

We collected information about the foster caregivers, including the caregiver's age, number of dogs in their home, the method by which the caregiver obtained their foster dog (i.e., placement of the animal inside the caregiver's vehicle, or the caregiver collecting the dog by coming inside or outside of the shelter), and whether the foster caregiver adopted their fostered dog. We also characterized the caregiver's relationship to the animal shelter. These foster caregiver roles were: (1) a member of the community who had not previously fostered for the organization, (2) a volunteer fostering for the first time, (3) a returning caregiver who fostered prior to the pandemic or more than once during pandemic, (4) a community member (role 1) fostering again, (5) a staff member, (6) a finder of a lost dog in the community who agreed to foster the dog, or (7) an owner rehoming their pet with the assistance of the shelter and continuing to care for their pet during that time. For the purposes of our study, finders and owners, who represented a very small portion of foster caregiving, were aggregated into a single category.

### Foster Utilization Ratio (FUR)

To understand the utilization of foster caregiving at each shelter and across our multi-shelter dataset, we calculated a Foster Utilization Ratio (FUR) by counting the number of dogs and puppies in foster care each day divided by the daily number of dogs and puppies with foster caregivers and those in the shelter combined. To determine the number of dogs in foster care on any given day, shelters' data collection spreadsheets were used to tally the number of dogs currently recorded as being in foster care. To establish the number of dogs living in the shelter, daily inventory and population reports were used. (Thomasville–Thomas County Humane Society was not included in this analysis as their recordkeeping did not allow for this type of data to be collected on a daily basis.) When discrepancies were found in a shelter's records about a dog's location, we used other methods to resolve those inconsistencies, including investigating database records and conversations with staff. A shelter's monthly FUR reflects the monthly average of their daily FUR.

### Statistical Analysis

Because this study was a natural experiment in its design, we used chi-square goodness of fit tests to test for distribution differences among various shelter, dog, and foster caregiver demographic variables; and Pearson correlation tests to measure the linear relationships between dogs' length of stay and adoption procedures. Test assumptions were checked through descriptive statistics.

With chi-square analyses, all cells contained at least one observation, and 80% or more contained at least five observations. In cases where cells contained less than five observations, categories were either combined with other logically consistent categories or removed from the analysis if the categories had consistently low counts. When conducting correlational analyses, variables were reviewed for normality and outliers. With the exception of dogs' total length of stay, no substantial outliers were found. Outliers in dogs' total length of stay were verified but remained in the dataset.

A multiple linear regression analysis with backward elimination was used to determine whether a shelter's average foster utilization could be predicted from its organization type, admissions policy, budget, canine intake, live release rate, or canine length of stay. Dummy variables were created for all categories within the variables of organization type and admissions policy, except for private non-profit and open admission, as these were the largest groups within these predictors and were used as the comparison groups.

To test whether FUR values differed across time, by organization type, or in an organization-by-month interaction, we analyzed shelters' FUR values with a linear mixed model. Shelter and intercept were entered as random effects. Month, organization type, and an organization-by-month interaction along with the covariate of the previous year's average length of stay for dogs were entered as fixed effects. (The factor of organization type and covariate of average length of stay were identified in the multiple linear regression analysis.) A variance covariance matrix was employed, and a diagonal covariance matrix for the repeated time point measure. The method of Restricted Maximum Likelihood (REML) was used for estimating parameter values.

### Ethical Statement

Study procedures were approved by the Arizona State University Institutional Review Board (STUDY:00008751).

## Results

### Shelter Demographics, Processes, and Procedures

Data were collected from 19 animal shelters across the United States. Shelters differed in their geographic location, admission policy, organization type, number of dogs brought into their facilities the previous year, and number of dogs and foster experiences that they contributed to the dataset (Table 2). Four animal shelters concluded data collection earlier than June 30, 2020: Carroll County Animal Services, Thomasville–Thomas County Humane Society, and Roice–Hurst Humane Society ended on June 29, 2020; and City of Irving Animal Services on June 26, 2020. (The municipalities where these shelters were located lifted their stay-at-home orders prior to June 30, 2020).

**Table 2:** Location of animal shelter, organization type, admission policy, 2019 canine intake, and number of dogs fostered & foster experiences recorded (March 11–June 30, 2020).

**Shelter demographics**				**Study participation**		
**Shelter**	**State**	**Organization type**	**Admission policy**	**2019 canine intake**	**Dogs fostered**	**Foster experiences**
Animal Care Sanctuary	PA	Pnp	Limited	387	28	31
Carroll County AS	GA	Municipal	Open	2471	29	29
Irving AS	TX	Municipal	Open	2650	30	32
Stockton AS	CA	Municipal	Open	6374	79	88
Good Shepherd HS	AR	Pnp	Limited	121	15	21
HS of Pinellas	FL	Pnp	Managed	1386	84	102
HS of Wicomico County	MD	Pnp+Contracts	Open	795	68	76
Nashville Humane Association	TN	Pnp	Limited	2604	414	603
New River HS–Fayette County ACC	WV	Pnp+Contracts	Open	1025	109	115
Pasadena HS & SPCA	CA	Pnp	Open	3659	89	101
Pets in Need	CA	Pnp+Contracts	Open	825	63	140
Roice–Hurst HS	CO	Pnp	Open	424	47	52
Sand Springs Animal Welfare	OK	Municipal	Open	531	18	18
Souris Valley AS	ND	Pnp	Managed	252	13	13
St. Hubert's Animal Welfare Center	NJ	Pnp	Managed	4363	197	238
Thomasville–Thomas County HS	GA	Pnp+Contracts	Open	1153	62	74
Wadena County HS	MN	Pnp	Open	560	95	117
Wisconsin HS	WI	Pnp	Open	4841	112	128
Young–Williams Animal Center	TN	Pnp+Contracts	Managed	4566	431	597

*Shelter abbreviations: AS, Animal Shelter; HS, Humane Society; ACC, Animal Control Center; SPCA, Society for the Prevention of Cruelty to Animals. Organization type abbreviations: Pnp, Private non-profit; Pnp+Contracts, Private non-profit with municipal contracts*.

Over three-quarters (78.95%) of animal shelters were private, non-profit organizations with one-third of these organizations fulfilling contracts with neighboring municipalities. Nearly two-thirds of shelters (63.16%) were open admission facilities with the remaining either managing (21.05%) or limiting (15.79%) their admissions. All shelters except one had some sort of dog fostering program prior to the pandemic.

For the year prior to the pandemic, live release rates (LRR) for dogs varied across shelters, with an average rate of 93.66% (*SD* = 9.61) with a range of 67.00 to 99.80%. Shelters' annual operating budgets for 2020 ranged from $100,000 to $12,000,000 (*M* = 2,840,497, *SD* = 3,733,186). Their mean yearly animal intake for 2019 was 3,956 animals (*SD* = 4,313) with a range of 223 to 16,357 animals. By using annual operating budgets and intake of animals, we estimated each shelter's available resources on a per animal basis. Based on this calculation, shelters were categorized into five resource groups, due to clear breaks in the resource ranges: very low ($116–207/animal), low ($304–396), moderate ($557–734), high ($837–990), and very high ($1,547 −2,305). Table 3 describes the average and median operating budgets and annual intakes by resource group, including the count of shelters and animals within each group.

**Table 3:** Shelter resource levels and associated annual budgets, animal intake numbers, and resources per animal.

**Resource** **level**	***M, Mdn* Annual** **budget**	**Annual budget range** **(Min–Max)**	***M, Mdn* 2019** **Animal intake**	**2019 Animal** **intake range** **(Min–Max)**	***M, Mdn* Resources** **per animal**	**Resources per** **animal range** **(Min–Max)**	**Shelters**	**Animals**
Very Low	973K, 538K	100K−2.71M	2341, 1743	862–4418	162, 162	116–207	4	186
Low	1.32M, 1.52M	250K−3.70M	3526, 2018	726-9344	353, 355	304–396	4	304
Moderate	6.40M, 5M	2.20M−12M	9605, 8971	3486–16357	636, 618	557–734	3	957
High	2.30M, 668K	391K−6M	2502, 1591	467–6797	928, 954	837–990	5	404
Very High	4.66M, 1.6M	370K−12M	2891, 694	223–7575	1837, 1659	1547–2305	3	132

*M, Millions; K, Thousands. Annual budget and resources per animal and their associated ranges are in US$. Resources per animal is an estimated value calculated by dividing a shelter's annual budget by the previous year's number of animals brought into the facility*.

Concerning the processes and procedures used by shelters, slightly more than half were conducting routine behavioral assessments with their dogs (52.63%), and 57.89% of organizations were assessing dogs' abilities to interact with other dogs. Of the shelters that were conducting dog-to-dog assessments, 45.45% were conducting them one-on-one with another dog, another 45.45% used a combination of one-on-one and group interactions, and the remaining shelter was assessing dog skills while out in groups with other dogs. More often than not, organizations did not have behavior personnel on staff. During the first 4 months of the pandemic, nearly two-thirds of shelters (63.16%) stopped or reduced the number of spay-neuter surgeries they were performing, and over a quarter (26.32%) were not requiring spay-neuter prior to adoption. Table 4 describes these processes and procedures, including counts and percentages of shelters in each category.

**Table 4:** Behavior and veterinary processes and adoption procedures undertaken by shelters during the COVID-19 pandemic.

	**Shelters**	**% (of** **shelters)**
**BEHAVIOR AND VETERINARY PROCESSES**		
**Behavior**		
Routine behavioral assessment	10	52.63
Dog-dog assessment	11	57.89
*One-on-one with another dog*	*5*	*45.45*
*Combination of one-on-one & group interactions*	*5*	*45.45*
*Group interactions*	*1*	*9.09*
Behavior personnel on staff	8	41.60
**Veterinary**		
Stopped or reduced number of spay-neuter surgeries	12	63.16
Reduced number of in-house veterinarians	7	36.84
Reduced partnerships with outside veterinary clinics	3	15.79
Did not require spay-neuter surgery before adoption	5	26.32
**ADOPTION PROCEDURES**		
**Meeting requirements**		
Humans in the household	2	10.53
Dogs in the household	4	21.05
**Meeting location**		
Meet at shelter	16	84.21
Meet at foster caregiver's home	8	42.11
**Paperwork location**		
Completed at shelter	18	94.74
Completed with foster caregiver	5	26.32
Completed with shelter, remotely	15	78.95
Completed with foster caregiver, remotely	1	5.26
**Adopted dog pick-up**		
Inside the shelter	18	94.74
Drive-through, at shelter	10	52.63
At foster caregiver's home	11	57.89

*All bolded categories include processes and procedures that are not mutually exclusive, except for dog-dog assessment. Shelter staff conducted these assessments either one-on-one with another dog, in group interactions with multiple dogs, or used a combination of both methods. The associated percentage of shelters is reflective of only those conducting dog-dog assessments*.

With regards to shelter adoption procedures during the early months of the pandemic, nearly 90% of organizations did not require all family members to meet the dog prior to adoption, and only 21.05% of shelters required meetings between any resident dog(s) and the shelter dog. In fact, over three-quarters of shelters (78.95%) had no meeting requirements whatsoever prior to adoption. As for the meet-and-greet venues, 84.21% of organizations were conducting meetings between adopters and dogs at the shelter, and 42.11% of shelters had foster caregivers handling meet-and-greets with potential adopters. Nearly all shelters (94.74%) were processing adoption paperwork at the shelter with the adopter, and 78.95% were processing it online with their adopters. Almost one quarter (26.32%) of shelters had foster caregivers handling the adoption paperwork in person with the adopters of their fostered dogs, and only one shelter had foster caregivers handling the paperwork online with them. Except for one shelter, all facilities were open for adopters to pick up animals inside their buildings. Over half of the shelters (52.63%) utilized a drive-through method of placing adopted animals directly in adopters' vehicles without the adopters coming into the shelter, and 57.89% were allowing adopters to pick-up their dogs directly from the foster caregiver's home (Table 4).

Between March 11 and June 30, 2020, 1,155 dogs and 323 puppies were placed into foster care, and 747 dogs were fostered by potential adopters at 19 animal shelters for a total of 2,225 animals. Most of these animals entered their shelters as transfers from another facility (40.18%). Over a quarter arrived to the shelter as a stray (28.42%), and one fifth were surrendered by their owner or were a failed adoption (20.25%). Males and females were practically equally represented (males: 50.78%). Excluding puppies, dogs were slightly over 3 years of age at the time of entering foster care (*M* = 38.47 months, *SD* = 36.16) and weighed, on average, 17.64 kg (*SD* = 10.43). Since dogs could be fostered more than once during data collection (such as multiple foster experiences during a single shelter stay or across multiple shelter stays), we also collected information about their individual foster experiences. Overall, dogs and puppies had 1,331 and 371 foster experiences, respectively, and there were 869 foster-to-adopt experiences for a total of 2,571 foster experiences.

### Foster Caregivers and Their Experiences

When describing the caregivers that provided fostering, 39.60% were new caregivers in the community, fostering for this shelter for the first time. Almost five percent (4.88%) were already volunteering for the organization but had never fostered prior to the pandemic. Over a third of foster caregivers (34.49%) had previously fostered for the organization, and 12.81% were new caregivers that started fostering during the pandemic and returned to foster again. Over seven percent of caregivers (7.29%) were staff, and less than one percent were finders and owners fostering dogs. Table 5 provides the foster caregivers and their relationship to the shelter by foster type. For statistical analysis, caregivers were further categorized as having a relationship (or not) to the shelter. Those individuals considered to have a prior relationship included staff, returning foster caregivers, shelter volunteers who were fostering for the first time, and new caregivers that began fostering during the pandemic but returned to foster more than once. (Dogs that were fostered by the finder or owner were excluded from relationship analyses. The incidence of these fostering situations was quite rare, and it was unclear whether these individuals had a preexisting relationship with the shelter).

**Table 5:** Foster caregivers and their relationship to shelter by fostering type and number of resident dogs living in the home.

			**Number of resident dogs**				
			**(% of foster caregivers)**				
**Caregiver's relationship** **to the shelter**	**Fostering type**	***n***	**0**	**1**	**2**	**3**	**4+**
**No prior relationship**							
New community member	Puppy	52	71.15	21.15	5.77	1.92	–
	Dog	622	74.60	18.33	5.14	1.29	0.64
**Prior relationship**							
Shelter volunteer*	Puppy	16	62.50	18.75	12.50	6.25	–
	Dog	67	70.15	19.40	4.48	5.97	–
Returning community member**	Puppy	72	80.56	12.50	6.94	–	–
	Dog	146	69.86	15.75	4.11	2.74	7.53
Returning foster caregiver	Puppy	187	28.88	15.51	27.27	17.11	11.23
	Dog	400	43.75	29.00	11.00	12.00	4.25
Staff	Puppy	44	–	34.09	20.45	20.45	25.00
	Dog	80	10.00	20.00	13.75	27.50	28.75
							
Finder/owner	Puppy	0	–	–	–	–	–
	Dog	16	43.75	18.75	18.75	–	18.75
**Overall**		1702	56.52	20.68	9.93	7.58	5.29

Puppy fostering is the caregiving of puppies that are under eight weeks of age when fostering commences. *Shelter volunteers are foster caregivers that volunteered at the shelter but had not previously fostered. **Returning community members are new community members that fostered again. The category of finder/owner was excluded from relationship analyses.

The average age of foster caregivers was 36.01 years old (*SD* = 13.07). We found that the presence of a dog was not equally distributed across foster types, *X*^2^(1, *N* = 1686) = 196.84, *p* < 0.0001. Puppy caregivers were more likely to have resident dog(s) in their home (57.14%) as compared to adult dog foster caregivers (39.67%). Additionally, if a foster caregiver of either type was dog-owning, the caregiver most often had one dog (47.57%) followed by two (22.84%), and three (17.43%), and the smallest proportion of homes were those that had four or more dogs (12.16%). This pattern of fewer foster caregivers as the number of resident dogs increased in the home was consistent for both puppy and dog caregivers (Table 5).

During the pandemic, novel approaches to foster animal pickup were implemented in an effort to increase social distancing and reduce the spread of the coronavirus. The most common approach included a drive-through style in which caregivers remained in their vehicles, and shelter staff placed foster animals inside. Around one third of foster experiences (36.13%) began this way. A further third (31.73%) were more typical, with the caregiver going inside the shelter to collect their foster animal. Almost one quarter of pickups were conducted outdoors with shelter staff (28.38%), and 3.76% occurred in some other way (i.e., foster caregiver swap, delivery of dog by the shelter to the foster home, or if a finder of a lost dog became its foster caregiver).

To understand whether resources influenced the types of caregivers that were fostering at these organizations, we tested whether relationship type (those with and without a prior relationship to the shelter) and individuals that fostered with the intention to adopt, were equally distributed among shelter resource levels. We found that the types of foster caregivers differed significantly by resources (*X*^2^(16, *N* = 1719) = 160.12, *p* < 0.0001). Very low-resource shelters utilized more caregivers with prior relationships to their organizations during the pandemic, representing 78.79% of all foster experiences. Conversely, the largest proportion of foster caregivers at the highest resourced shelters were new foster caregivers from the community (60.63%). Lastly, moderately resourced shelters showed a far higher rate of foster-to-adopt arrangements (43.77% of foster experiences) versus the next closest foster-to-adopt rate demonstrated by high resource shelters (32.84%).

Approximately a fifth of foster experiences were with dogs that had additional behavioral needs when they entered foster care. Puppies needing behavioral management were virtually absent (0.62%). Dogs and puppies needing medical management represented 32.90 and 21.67%, respectively, of foster experiences. Bites to a person or animal rarely occurred. Only 15 bites (1.1% of dog foster experiences) were reported, with a roughly even split amongst incidents involving other dogs (seven bites) and people (eight bites). Additionally, bites were more often inflicted by dogs without known behavioral concerns (66.66%) compared to those that did (33.33%).

We found the reason that foster care ended significantly differed by foster type (*X*^2^(5, *N* = 686) = 141.02, *p* < 0.0001). Not surprisingly, puppies most often had a scheduled return to the shelter (44.74%), likely due to their age (i.e., reaching 8 weeks) and a change in availability, followed by a return for adoption (39.62%), with 11.33% returning to the shelter due to issues related to their caregiver. The majority of adult dogs (62.43%) left foster care because of a potential adoption, with 16.30% having the return previously scheduled by the shelter, and 9.02% returning to the shelter for behavioral issues. Returns of fostered dogs that were caregiver-related comprised 10.15% of foster experiences (Table 6).

**Table 6:** Proportion of foster care returns by reason and fostering type.

**Reason foster care ended**	**Puppy**	**Dog**
Adoption	39.62	62.43
Behavioral	1.08	9.02
Medical	3.23	2.10
Caregiver-related	11.33	10.15
Scheduled return by the shelter	44.74	16.30

We also examined whether dogs that were sent out to foster care in need of medical or behavioral management were returned by their foster caregivers for a similar issue. We found 49 out of 288 behaviorally managed foster experiences ended for behavioral reasons, representing 17.01% of all foster experiences that needed behavioral management. Conversely, dogs without behavioral issues were returned in 5.30% of foster experiences, and this difference in returns between dogs with and without behavioral management was statistically significant, *X*^2^(1, *N* = 1,702) = 48.57, *p* < 0.0001. We found that 23 out of 508 foster dogs needing medical support were returned because of medical issues, representing 4.53% of all medically managed foster experiences. This is significantly more than the 1.42% of non-medically managed experiences in which dogs were returned (*X*^2^(1, *N* = 1,702) = 14.96, *p* = 0.0001). Though statistically significant, its practical significance may be limited.

### Fostered Dogs: Outcomes and Length of Stay

Over 93% of fostered dogs and puppies had positive outcomes: 83.35% were adopted to new owners, most often directly from the foster caregiver's home or from the shelter with just a very short time at the facility prior to pick-up. Transfers to other agencies for placement constituted 9.45% of outcomes, and a minuscule percentage (0.27%) of dogs were returned to their owners. More puppies were transferred out (15.02%) than adult dogs (7.82%). At the end of the study, slightly over five percent of both dogs and puppies remained in the care of their organizations, either in a foster home (4.15%) or at the shelter (1.22%). Less than two percent of all fostered animals had negative outcomes: 0.34% were lost in care (and unable to be found), 0.20% died in care, or were euthanized for behavioral (0.54%) or medical (0.48%) reasons; however, no puppies were euthanized for behavior.

Combining both time in the shelter and foster care for an animal's total length of stay, dogs (both those fostered by potential adopters and foster caregivers) and puppies spent an average of 43.35 days (*SD* = 51.49, *Mdn* = 31.00, IQR = 40.00) in the care of their organization. When considering separately the duration of that time that was spent in foster care, these animals were fostered an average of 19.52 days (*SD* = 23.99, *Mdn* = 11.00, IQR = 21.25) with a range of 0 to 176 days. A value of zero for length of stay refers to someone who cares for a dog they found that is then reunited with its owner within the same day. Additionally, we found that age was positively correlated with adult dogs' length of stay (*r* (1,702) =0.124, *p* < 0.0001), such that as a dog's age increased, so did their time in foster care.

When examining dogs' lengths of stay, we found that the number of days spent in foster care was not uniformly distributed across foster type (*X*^2^(1, *N* = 1,686) = 131.22, *p* < 0.0001). Dogs that left the shelter with potential adopters remained in their care for an average of 16.18 days (*SD* = 26.60, *Mdn* = 6.00, IQR = 14.00) while fostered dogs spent 20.93 days (*SD* = 22.68, *Mdn* = 13.12, IQR = 22.15) and puppies, 22.29 days (*SD* = 22.17, *Mdn* = 16.98, IQR = 22.02) in foster care. Furthermore, this duration in foster care differed if the dogs were adopted by their potential adopter or foster caregiver. Adopted foster-to-adopt dogs spent 15.74 days (*SD* = 23.62, *Mdn* = 7.00, IQR = 16.62) with their potential adopter and 17.37 days (*SD* = 33.36, *Mdn* = 3.00, IQR = 12.75) if the person did not adopt. Dogs that were fostered by a caregiver who adopted them, spent on average 28.58 days (*SD* = 25.21, *Mdn* = 21.08, IQR = 26.02) in foster care, and 19.61 days (*SD* = 21.16, *Mdn* = 11.94, IQR = 20.58) when the caregiver did not adopt; while puppies were fostered for an average of 41.61 days (*SD* = 33.13, *Mdn* = 34.71, IQR = 29.03) if their caregivers adopted them, and only 20.77 days (*SD* = 19.20, *Mdn* = 16.14, IQR = 20.20) if they did not.

We found that the lengths of stay of dogs and puppies were not uniformly distributed across surgery timing categories, *X*^2^(5, *N* = 1,471) = 17.04, *p* =0.004. As expected, dogs that arrived already spayed or neutered had the shortest length of stay, on average, with 43.59 days (*SD* = 52.03, *Mdn* = 28.50, IQR = 41.75). For puppies, their length of stay was shortest when altered after adoption. Conversely, dogs that were altered during foster care had, on average, the longest length of stay (90.66 days, *SD* = 241.94, *Mdn* = 45.50, IQR = 49.00). Puppies had the longest lengths of stay when they were spayed or neutered during their time with a foster caregiver. Table 7 provides the counts of fostered dogs and puppies and associated lengths of stay in each of the surgery timing categories.

**Table 7:** Length of stay (in days) by timing of spay-neuter surgery and fostering type.

**Timing of spay-neuter surgery**	**Fostering type**	***n***	***M***	***SD***	***Mdn***	**IQR**
Before intake to the shelter	Puppy	1	38.00	–	38.00	–
	Dog	443	43.59	52.03	28.50	41.75
At the shelter	Puppy	99	37.36	20.24	38.00	36.50
	Dog	301	48.86	49.76	33.00	39.00
In foster care	Puppy	10	57.70	26.33	53.00	13.25
	Dog	62	90.66	241.94	45.50	49.00
During a foster-to-adopt	Puppy	36	56.08	27.45	50.00	34.00
	Dog	68	57.40	26.33	48.50	20.25
After leaving the shelter	Puppy	166	25.67	23.42	16.50	40.50
	Dog	184	60.04	92.72	25.00	47.25

*IQR, Interquartile range*.

In considering the associations between dogs' length of stay and various adoption practices used during the pandemic, many of these practices were often carried out (e.g., completing adoption paperwork at the shelter or remotely) or not carried out (e.g., required meetings for humans or dogs in the household) by a majority of participating shelters (see Table 4), creating the possibility that detected correlations may be more reflective of those shelters and less about the particular practice itself. With this in mind, we examined three practices in which shelters were equally or nearly equally split in their usage: allowing potential adopters to meet with foster caregivers, and two types of adoption pick-up methods, at the foster caregiver's home and drive-through at the shelter.

To identify potential relationships between these adoption practices and dogs' foster length of stay, we used Pearson correlation tests. (In these analyses, we excluded dogs that had behavioral or medical concerns that could have impacted their lengths of stay as well as dogs that were adopted by their foster caregivers.) We found that when shelters allowed potential adopters to meet fostered dogs at the caregiver's home, these dogs had shorter lengths of stay in foster care (*M* = 13.72 days, *SD* = 16.43, *Mdn* = 10.00, IQR = 18.80) compared to shelters that did not (*M* = 21.95 days, *SD* = 17.03, *Mdn* = 16.17, IQR = 23.20). This relationship was weakly but significantly correlated, *r* (1,825) = −0.206, *p* < 0.0001. We also found that when shelters allowed adopters to pick up their dogs directly from the foster caregiver, dogs at these shelters had shorter lengths of stay (*M* = 15.86 days, *SD* = 18.85, *Mdn* = 11.07, IQR = 22.55) compared to dogs at shelters that did not allow this type of pick-up (*M* = 20.83 days, *SD* = 14.45, *Mdn* = 15.88, IQR = 22.07). This was a weak yet statistically significant correlation, *r* (1,825) = −0.163, *p* < 0.0001. Lastly, dogs' lengths of stay at shelters that had a drive-through pick-up option were slightly longer (*M* = 18.20 days, *SD* = 14.33, *Mdn* = 14.76, IQR = 21.70) than dogs at shelters without this mode of acquisition (*M* = 17.77 days, *SD* = 20.74, *Mdn* = 11.73, IQR = 21.74). However, when we consider the conflicting nature of the test coefficient, *r* (1,825) = −0.171, *p* < 0.0001, in addition to these average lengths of stay and their large standard deviations, this finding is difficult to interpret.

**Figure 1:**
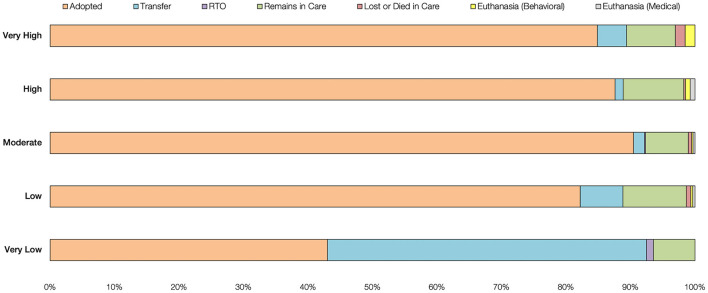
Proportions of outcome types by shelter resource level.

In order to examine the role that shelter resources may have played in outcomes, we tested whether the numbers of animals in the various outcome categories were uniformly distributed across our five shelter resource levels. We found differences in outcomes based upon the resource level of the organization (*X*^2^(4, *N* = 1,983) = 614.19, *p* < 0.0001). Specifically, very low resource shelters made substantially greater use of transferring animals out of their facilities (49.46%) than low or moderately resourced shelters (6.58 and 1.67%, respectively). Additionally, the adoption rate at very low resource shelters was 43.01%, while shelters at all other resource levels placed fostered dogs directly with adopters at rates above 80% (Figure 1).

We found that dogs fostered by their potential adopters (as in a foster-to-adopt) or by a foster caregiver with a dog or puppy had different likelihoods of adoption (*X*^2^(5, *N* = 2,555) = 902.00, *p* < 0.0001). Nearly three-quarters (72.96%) of foster-to-adopt experiences ended in adoption, while only 18.03% of dog and 7.28% of puppy foster experiences ended in adoption (Table 8). Thus, individuals that were fostering with the intention to adopt were, in fact, the most likely to adopt, yet dog foster caregivers were twice as likely to adopt their fostered dog as puppy caregivers.

**Table 8:** Adoption of fostered dogs and puppies by caregiver's relationship to the shelter.

**Caregiver's relationship to the shelter**	**Adopted** ***n***	**Did not adopt** ***n***	**Adopted** **(%)**
**No prior relationship**			
New community member	195	484	28.72
**Prior relationship**			
Shelter volunteer	12	70	14.63
Returning community member	18	194	8.49
Returning foster caregiver	31	558	5.26
Staff	8	116	6.45
Potential adopter (foster-to-adopt)	634	235	72.96

In further examining the outcomes of fostered dogs by the type of relationship the caregiver had with the shelter, we found that adoptions by foster caregivers were not uniformly distributed across relationship type (*X*^2^(1, *N* = 1,686) = 148.23, *p* < 0.0001). New caregivers with no prior relationship to the shelter adopted their fostered dogs at a rate of 28.72% while those with a relationship, such as returning foster caregivers, shelter volunteers, and staff, did so at a combined rate of 6.85% (Table 8). Additionally, we found differences in the likelihood of adoption by foster caregivers with and without a relationship to the shelter, dependent upon the number of dogs in their home (*X*^2^(4, *N* = 1,686) = 208.10, *p* < 0.0001). New caregivers without a relationship to the shelter or resident dogs in their home were more likely to adopt their fostered dog (77.39%) than new caregivers with any number of dogs in their home (22.61%). The same was true for foster caregivers with a prior relationship to the shelter with some differences. Those without dogs adopted most often (46.15%) as compared to those with one (30.65%), two (14.52%), or three or more dogs (9.68%).

### Foster Utilization Ratio (FUR)

A multiple linear regression analysis with backward elimination was used to identify whether a shelter's average foster utilization could be predicted from characteristics about the shelter, including the previous year's canine intake, live release rate, and length of stay as well as the shelter's organization type, admissions policy, and current year's operating budget. Two variables, including the shelter's 2019 length of stay for dogs 6 months and older and organizations that were public municipal agencies, remained in the equation and accounted for 39.90% of the variability in shelters' average foster utilization, *F* (2, 13) = 5.97, *p* = 0.014.

We found that the classification of the organization as a public municipal agency was significantly predictive of foster utilization compared to private non-profit shelters (β = −30.17, *p* = 0.011), such that the FURs of public municipal agencies were 30 points lower when compared to private non-profits. Additionally, shelters' 2019 canine length of stay trended toward predicting their foster utilization (β = −0.384, *p* = 0.073). This marginal finding would suggest that for each day that a shelter's 2019 length of stay was shorter, their FUR increased by slightly more than one-third of a point.

Using shelters' daily utilization of foster care for March through June 2020, we analyzed these values to detect an effect of month, organization type, or a month-by-organization-type interaction with shelters' 2019 average length of stay for adult dogs added as a covariate in the model based on the regression analysis. With this model, the variables of month, organization type, and the month-by-organization-type interaction were significant (at *p* < 0.05). The length of stay variable, however, was not significant in the model (*p* = 0.105) but was retained.

The main effect of month was significant, *F* (3, 1761.00) = 99.71, *p* < 0.001, indicating that foster utilization changed across time. We found in *post-hoc* comparisons that shelters had significantly higher utilization in April as compared to all other months (*p* < 0.001). May was also higher than March (*p* = 0.006) and June (*p* < 0.001), and lower than April (*p* < 0.001). June was not significantly different than March (*p* = 0.173). A main effect of organization type was also detected, *F* (2, 12.00) = 4.22, *p* = 0.042, signifying that the estimated marginal means for foster utilization varied across different types of shelters. In *post-hoc* comparisons, we found that public municipal agencies had the lowest foster utilization ratios (*M* = 14.52, *SE* = 9.04), however these organizations only marginally differed from private non-profits in their daily foster utilization (*p* = 0.058) and not from private non-profits that had government contracts (*p* = 0.140).

The interaction of organization-type-by-month was significant, *F* (6, 1761.00) = 19.62, *p* < 0.0001, indicating that shelters' daily FURs varied each month in different ways based on their organization type. When examining these organizational monthly differences in detail, private, non-profit shelters had significantly higher foster utilization in April and May as compared to March and June (*p* < 0.001). Very little change in foster utilization occurred for these shelters between April (*M* = 48.86, *SE* = 6.25) and May (*M* = 47.48, *SE* = 6.25). For private, non-profit shelters with municipal contracts, foster utilization in April was significantly higher than all other months (*p*< 0.032). However, May FUR was not significantly higher at private, non-profit shelters with municipal contracts than March foster utilization (*p* = 0.998), as was seen with private, non-profits.

**Figure 2:**
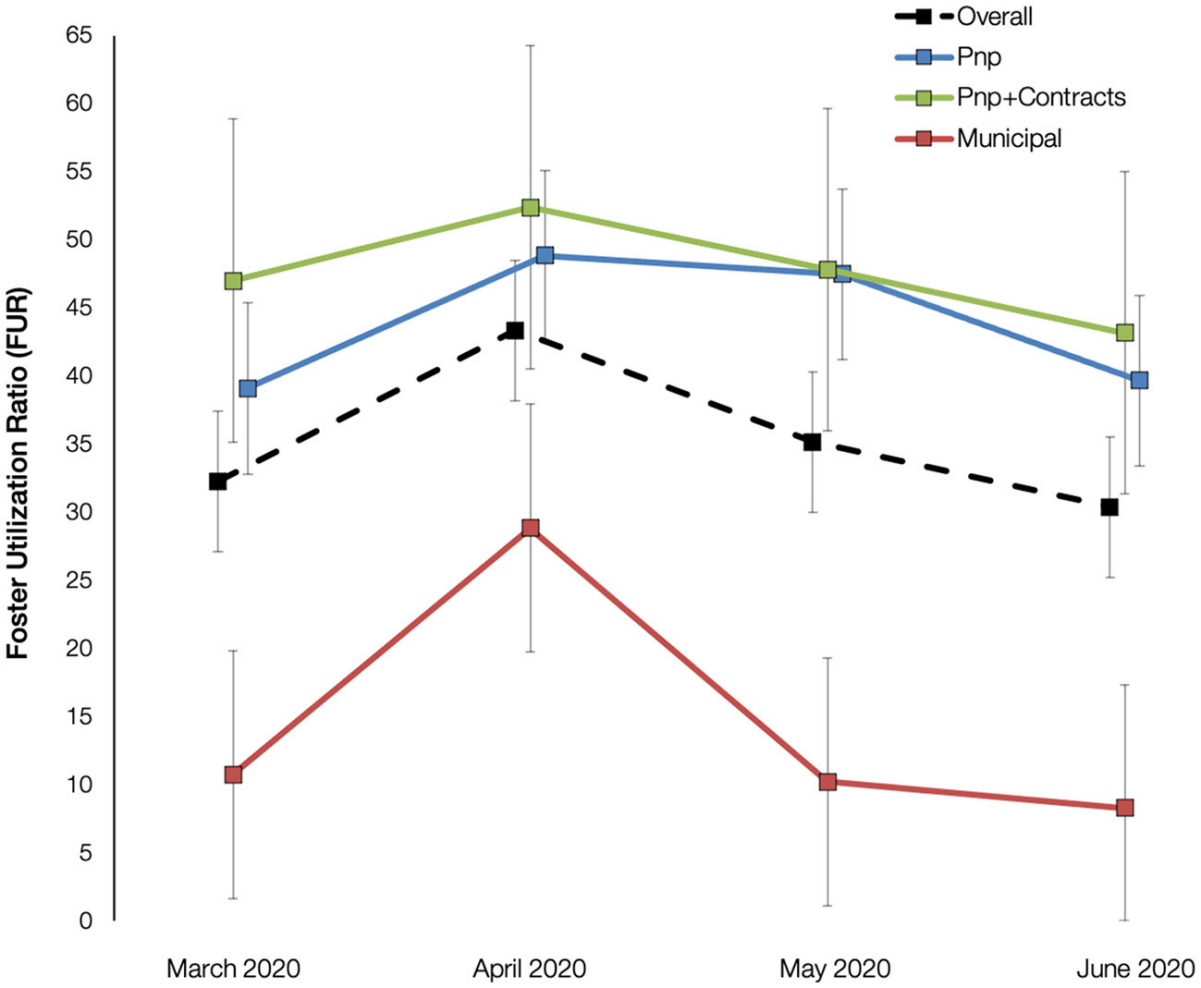
Estimated marginal means and standard errors of foster utilization by organization type from March96June 2020.

For municipal shelters, April was again the month of highest foster utilization compared to all other months (*p* < 0.001), but a return to FUR levels seen at the beginning of the pandemic (March *M* = 10.74, *SE* = 9.11) was already occurring in May (*M* = 10.22, *SE* = 9.08, *p* = 1.00). Moreover, this level of foster utilization in May by municipal shelters was significantly lower than utilization by private, non-profit shelters (*p* = 0.015) and trending lower compared to private, non-profit shelters with municipal contracts (*p* = 0.084). By June, FUR for municipal agencies had dropped to an estimated marginal mean of 8.27 (*SE* = 9.09), which was significantly less than private, non-profit shelters (*p* = 0.041). Figure 2 presents the estimated marginal means and standard errors of the foster utilization ratios at the three organization types from March through June 2020.

## Discussion

This investigation found that shelters' utilization of foster care during the COVID-19 pandemic increased from March to April 2020 but returned to initial levels by June 2020. This effect was more pronounced for municipal agencies (compared to both categories of private, non-profit shelters) in that they experienced similar April increases in foster caregiving, but their foster utilization returned to initial pandemic levels by May 2020. Additionally, we were able to characterize new variables, specifically a shelter's available resources and foster caregivers' relationships to the shelter, that explained the behavior of the animal welfare organizations and their caregivers' adoptive behavior.

### Shelter Resources

Our analysis of shelters' modes of animal placement showed that the behavior of the organization depended on the resources available to them. While shelters across resource levels had similar live release rates for their fostered dogs (between 93 and 100%), how shelters accomplished those outcomes varied. Shelters at every resource level except the very lowest resourced, used adoption as their main method of dog placement. Prior to the pandemic, Woodruff and Smith (1) found that private non-profit shelters were most likely to adopt out dogs directly from their facilities, more so than municipal shelters and even private non-profits with municipal contracts. In our study, all high and very high resource shelters were private non-profit organizations. During the pandemic, shelters submitting their data to the Shelter Animals Count database utilized adoption as their predominant placement approach, irrespective of resources (31).

The lowest resourced shelters in our study, a mix of municipal and private non-profit shelters with municipal contracts, did not use adoption as their primary approach when placing dogs. Instead, they used a combination of transfer and adoption programs, relying more heavily on the former than the latter. In fact, shelters with very low resources transferred dogs out of their facilities at a rate of nearly 50%, which was several times higher than that of better resourced shelters. Pre-pandemic, Woodruff and Smith (1) found that municipal and private non-profit animal shelters with municipal contracts, were also more likely to transfer dogs out of their facilities than were private non-profits. Transferring dogs out of the shelter to another organization reduces the number of days an animal is in the care of the originating shelter and can be a cost-effective strategy to achieve live outcomes (17).

Resources, or lack thereof, also played a role in the individuals that shelters engaged to provide foster caregiving during the pandemic. Very low and low-resource shelters relied more heavily on foster caregivers who had a prior relationship with the shelter. Volunteers are a valuable resource to animal shelters (32), and foster caregivers voluntarily care for animals in their homes. For shelters with minimal budgets relative to the number of animals that they serve, depending on known caregivers, a majority of whom that had previously fostered, was likely the most economical approach of administering an external caregiving program throughout this time.

Conversely, our highest resourced shelters relied more heavily on new community members for their foster caregiving. Choosing to utilize likely less experienced caregivers is a more expensive decision for organizations as it requires responding to their initial inquiries and providing new caregiver training and fostering support. Previous research has shown that high quality training of volunteers can be costly (33), and the onboarding of new volunteers and their management during a crisis can be labor-intensive (34). While nearly all shelters had some sort of dog fostering program prior to the pandemic, it is possible that highly resourced shelters recruited foster caregivers because they had not previously invested in adult dog fostering programs given the resources available to them at the shelter.

### Foster Utilization Ratio (FUR)

We observed significant changes in the number of dogs cared for in foster care, relative to those living in the animal shelter. Specifically, we found an escalation in foster utilization in April 2020, a month where shelters had 43% of their dogs in foster care, an increase of over 30% compared to the start of the pandemic. For the municipal shelters, the difference was much greater; foster utilization rose by 270% relative to March 2020.

In addition to higher levels of foster caregiving, April's higher FURs were likely related to fewer animals entering shelters. Animal welfare organizations submitting inventory data for April 2020 reported sharp decreases in the number of animals taken into their facilities, reaching the lowest monthly levels reported since 2019 (31). Based on how FUR is calculated, decreases in intake during April 2020 would reduce the number of animals cared for in the physical shelter, thereby increasing the proportion of foster caregiving relative to the total number of animals in the organization's care. Conversely, increases in intake, with more animals living in the shelter than foster homes, would decrease FURs. This may explain why shelters were unable to maintain the high levels of foster caregiving observed in April, and to a lesser degree, May. Data from Shelter Animals Count suggest that animal intake returned to levels comparable to or higher than those reported in the months leading up to the pandemic by May and June 2020 (31).

Another explanation for decreased foster utilization may be related to the caregivers themselves. Foster caregivers with no relationship to the shelter prior to the pandemic comprised over half of all caregivers, yet fewer than a quarter of these caregivers returned after their initial experience. Overall, we found that only 25% of foster caregivers, regardless of their relationship to the shelter, fostered more than once during our four-month data collection. While long stays in foster homes may explain an inability for caregivers to foster additional dogs, this was not the case. Dogs typically remained in foster care for less than 3 weeks with a total length of stay (shelter and foster care) of a month-and-a-half. Thus, it is possible that caregivers could have fostered another dog, particularly as reported increases in intake in May and June would have provided new opportunities. Dog adoptions by foster caregivers, particularly by new community members, may be one explanation for why new caregivers failed to foster again.

### Foster Caregivers

The rise of volunteerism during the pandemic was not a surprising response to such societal uncertainty. Having the opportunity to care for an animal in need and join the animal shelter's community of volunteers would be a way to increase one's social support (35). In their exploration of pets and mental health during the early months of the pandemic, Ratschen et al. (36) found that pet owners reported smaller declines in mental health and smaller rises in loneliness compared to non-pet owners, suggesting a potential social buffering effect of pet ownership. While foster caregivers did not own the dogs they were caring for and thus the mental health benefits may have been less pronounced, it is likely that this caregiving experience provided a much-needed distraction from the pandemic (37).

When examining foster caregivers' relationships to their animal shelter, we found that new caregivers from the community were much more likely to adopt their fostered dogs, over four times more likely than caregivers who had a prior relationship to the shelter. Additionally, we found that individuals that fostered a dog with an interest in adopting did adopt their dogs in nearly three-quarters of those instances. This increased propensity for adoption, particularly with new foster caregivers from the community and potential adopters fostering their dogs, provides new insights into trial adoption programs.

To our knowledge, a study by Normando et al. (14) provides the only empirical evidence regarding the use of trial adoptions at an animal shelter. In their study, they found 100% of 110 dogs in an Italian animal shelter were adopted by individuals who used a trial period before formalizing their decision. In the United States, published evidence about the impact of such programs on adoptions has been scant, although they are recommended by animal welfare organizations (38). Our findings indicate that foster-to-adopt programs more often result in potential adopters becoming the dogs' owners. Even if a decision not to adopt results in the dog's return to the shelter, previous research has shown that a few days away from the stressful shelter environment are beneficial to dogs' psychological wellbeing (6).

The higher rate of adoption by new foster caregivers suggests that these caregivers may be more similar to potential adopters fostering with the intention to adopt than traditional foster caregivers. When we weigh the costliness of training new volunteers (33), animal shelters may be better served regarding first-time foster caregivers as individuals fostering with the potential to adopt. Not only would this approach reduce the consumption of resources involved in the onboarding of new caregivers (39), it would likely result in faster placement of dogs into caregivers' homes, thereby reducing their time in the shelter, providing additional cost savings to the organization (40). Consequently, it would appear that foster-to-adopt programs are an evidence-based best practice that improve both the proximate and distal welfare of shelter dogs.

While dogs that were fostered by potential adopters were most often adopted by those individuals, we found that traditionally fostered dogs were adopted by their caregivers in nearly one-fifth of foster experiences. Only 7% of puppy foster experiences ended in adoption by the caregiver. These dogs' lengths of stay in foster care varied, however, depending upon (1) whether a potential adopter or foster caregiver was providing the care and (2) whether the caregiver decided to adopt. Dogs that were fostered by potential adopters had shorter lengths of stay than dogs or puppies in traditional fostering programs. Furthermore, when potential adopters adopted, those dogs' lengths of stay were shorter than when they did not.

One possible explanation for this speedier decision-making from potential adopters, specifically from those that adopted, is that these individuals were already contemplating adoption. They had chosen a dog and were taking it home on a trial basis to gather more information. Shelters that allow adopters to foster prior to formalizing their decision may be reducing the perceived risks associated with adoption, allowing would-be owners to focus on the benefits of adding a dog to their household. Lenient return policies in the retailing literature have been shown to increase purchasing as well as return behavior of consumers (41). This would likely explain the high rate of conversion to adoption in nearly three-quarters of foster-to-adopt experiences as well as the increased rate of return compared to more traditional adoption programs (42). It is unclear, however, what impact fostering prior to adoption has on overall adoption rates.

Conversely, lengths of stay for both puppies and dogs that were adopted by foster caregivers were significantly longer than those that were placed with adopters. A possible explanation for the additional time that these dogs and puppies spent in foster care may be related to the caregivers changing their minds. Unlike potential adopters who foster because they are interested in adopting, caregivers typically foster with no declared interest in adopting. If their intentions changed during their foster experience, they may have needed additional time to arrive at those decisions.

It is likely that pet ownership, including newly adopted and other resident pets, influences foster caregiver retention and recruitment. In previous research, caregivers most often indicated that the needs of their own pets and adoption of previously fostered pets were reasons why they were no longer participating in foster programs (43). We found that the proportion of foster homes with resident dogs was slightly higher than the estimated percentage of canine-owning households in the US (44). In our study, over 56% of foster homes were without dogs, and those that were canine-owning most often had just one dog. It is possible that the acquisition of new dogs by foster caregivers and those fostering with the intention to adopt, may explain reduced foster utilization in May and June 2020. While the pandemic may have aided shelters in the recruitment of new foster caregivers and the adoption of shelter dogs (45), it is unclear whether it had the same effect on the retention of foster caregivers.

Despite the positive mental health benefits of foster caregiving during the pandemic (35–37), it is worth noting that animal fostering is a form of high stakes volunteerism (46, 47). Fostered pets, just like those that are owned, need supervision and daily husbandry, and caregivers develop strong emotional relationships with their animals. Recent research by Thielke and Udell (48) found that fostered dogs form secure attachments to their caregivers at similar rates to owned dogs. For the shelters that encourage foster caregivers to assist in adoption promotion and placement, caregiving may involve communicating and meeting with potential adopters. Considering the physical and emotional commitments involved, it is possible that new caregivers did not return to foster another animal because of a mismatch between their expectations and the reality of the position (49).

### Fostered Dogs

The outcomes for dogs were overwhelmingly positive with over 93% of dogs being either adopted, returned to their owners, or transferred to other agencies. Less than six percent of animals remained in the care of their organizations at the end of data collection, and more than three-quarters of those dogs were doing so in a foster home. Just over one percent of fostered dogs were euthanized for medical and behavioral issues, and only one fifth of one percent died in care. Live release rates of fostered dogs during the study were slightly higher than shelters' 2019 live outcomes for all dogs. We also found that dogs' length of stay in foster care was related to their age, such that as age increased, so did time in foster care. While this relationship has been observed with dogs awaiting adoption in the shelter (50, 51), this adopter preference for younger dogs in foster care has not been previously characterized.

Crowe and Patronek (17) found similar evidence of a positive relationship between foster care and live outcomes for shelter dogs. They found that the likelihood of live release for fostered dogs was over five times higher than that of stray and owner-surrendered dogs that did not experience foster care. In their study, dogs were often placed in foster care needing additional medical or behavioral treatment, while less than 15% of non-fostered dogs, that were adopted directly from the shelter, had behavioral or medical concerns. Taken together, our findings offer further evidence that foster caregiving is a worthwhile intervention for promoting the ultimate welfare of shelter dogs.

We identified that 21% of foster experiences included dogs that needed some sort of behavioral management, and roughly one-third of dogs required medical treatment during foster care. While more dogs entered foster care needing medical support, returns for medical-related issues with these dogs occurred in less than 5% of cases (compared to under 2% of returns for non-medical foster experiences). Yet, dogs needing behavioral management by their foster caregivers were returned three times more often for behavioral issues (17%) than non-behaviorally managed dogs (5%). Based on these findings, we would suggest that more specialized assistance for dogs entering foster care with known behavioral issues is needed.

Dogs with behavioral concerns prior to adoption placement have also been shown to have higher rates of return to the animal shelter. Recently, Friend and Bench (52) found that dogs displaying aggression to other dogs, when also factoring in their breed and size, have a greater risk of adoption failure. In a prior study exploring the use of behavioral assessments in the shelter, dogs that stiffened or growled during assessment were more likely to be returned by their adopters for behavior-related issues (53). However, the presentation of problem behaviors in the shelter does not necessarily predict similar behavioral issues in the home. Clay et al. (54) found that friendly and fearful behaviors that dogs displayed in a shelter assessment were also observed by adopters in the home. However, other behaviors, such as those related to separation and aggression, were not.

Still, it appears that animal shelters should consider providing support to adopters and foster caregivers of behaviorally-challenged dogs. In a review of adoption and relinquishment of dogs in the animal shelter, Protopopova and Gunter (42) identified that successfully supporting new adopters may involve providing more than general behavior advice or short counseling sessions, particularly if the dog's behavioral issues are more complex. Yet little in the scientific literature has described or experimentally tested these types of behavioral interventions (55, 56). Nevertheless, the returns of behaviorally managed fostered dogs in our study and adopted dogs due to behavior issues found by Hawes et al. (56), indicate a need for additional post-placement assistance to foster caregivers and adopters of these dogs.

### Shelter Processes

Differences in the timing of spay-neuter surgeries impacted length of stay. Puppies' lengths of stay were shortest when they were spayed or neutered after adoption compared to performing the surgery while in the shelter or in foster care. Thus, simply removing the need to sterilize while in the organization's care resulted in the shortest stays. Furthermore, puppies and dogs whose spay-neuter surgeries occurred any time during foster care had the longest lengths of stay. It is possible that the logistics of sterilizing animals that no longer reside in the shelter may have contributed to prolonged stays with these organizations.

The surgical sterilization of pets prior to adoption placement is a standard practice in US animal shelters to control the number of unwanted animals in communities. By altering soon-to-be-adopted animals before they are owned and living in homes, the spay-neuter procedure is assured to be completed (57). Our results suggest that animal shelters would reduce their lengths of stay for underage puppies by placing them in adoptive homes as quickly as possible and scheduling spay-neuter surgeries post-placement. (Most puppies in our study were not fostered as part of a litter, so this suggestion does not consider the behavioral benefits of fostering with other littermates until 8 weeks of age.) This arrangement, however, would likely lead to low compliance amongst adopters in the sterilization of their dogs (58).

Alternatively, shelters could place puppies in their adoptive homes as fostered dogs with owners who intend to adopt. Although this timing of spay-neuter resulted in the longest lengths of stay, it would likely ensure higher compliance with follow-up sterilization appointments if the adoption was formalized post-surgery (59) and achieve a similar result that adoption prior to spay-neuter surgery accomplishes: reducing the need for placement in a foster caregiver's home and acclimating the puppy sooner to the environment in which it will be living. Previous research has indicated the behavioral benefits of early exposure to people, objects, and experiences for dogs (60) while the effects of pediatric and early neutering on canine physical and behavioral health have become points of debate within the veterinary community (61).

Some of the adoption practices that we compared provide insights into ways in which animal shelters may be able to reduce length of stay in foster care. We caution that because this study was a natural experiment, we could not use random assignment and other techniques customary in experimental designs to control for dog-related variables that may have influenced the results. Furthermore, coefficients of these correlational tests range from very low to low, suggesting a likely small influence, if any, on dogs' length of stay. Thus, we report these findings for future studies to explore.

At the onset of the pandemic, animal shelters were encouraged to implement a variety of practices to accelerate placement into foster and adoptive homes, including caregiver-facilitated meet-and-greets and adoption directly from foster care (62). In our examination of these types of practices, we found that shelters that allowed foster caregivers to meet with potential adopters and adopters to pick up dogs from their foster caregivers, had dogs with shorter lengths of stay than shelters where these practices were not in place.

Practices which allow foster caregivers to interact with adopters and direct placement decisions could be described as a new form of open adoptions (63). Originally discussed in an American Humane Association forum in 1999 (64), open adoptions are a less restrictive approach to animal placement that encourage conversations between sheltering staff and potential adopters to inform placement decisions. In the two decades since this forum, open adoptions have become the predominantly recommended approach in animal sheltering (11, 65, 66).

Enabling foster caregivers to perform adoptions relies on the knowledge of caregivers and experiences with their fostered pets to inform placement decisions. Thus far, foster caregiver-directed adoptions have been shown to provide potential adopters with more useful information about the fostered dog and its behavior in a home and led to lower return rates when compared to dogs adopted from the animal shelter (15). While our correlational data would also support foster caregivers' involvement in the adoption process, future studies are needed to compare these types of foster caregiving practices and those by Mohan-Gibbons et al. (15) to more traditional forms of fostering without adoption components.

### Limitations

When considering the limitations of our study, it is likely that not all dogs at our participating shelters were made eligible for placement in foster care or had a caregiver who was interested in providing foster care due to various behavioral or medical issues. As we have identified in previous studies (6, 12), dogs with aggression issues are often not selected by staff for interventions with volunteers or members of the community. This safety bias may have led to fostered dogs having better outcomes, including higher live release rates, than shelters' 2019 data which included all dogs under the shelter's care. Despite this potential preference, dogs with behavioral and medical issues were still represented in the dataset.

While every effort was made to enroll animal shelters of various organization types, private non-profit organizations were over-represented in our dataset when compared to municipal shelters and, to a lesser degree, private non-profit shelters with municipal contracts. To address these shortcomings, we conducted analyses, not only at the organizational level, but created a shelter resource variable to better understand how monetary assets, relative to the number of animals a shelter served, influenced organizational decision-making. Nevertheless, private non-profit shelters accounted for all instances of very high and high resource shelters. The inclusion of more shelters with smaller budgets and/or more animals cared for annually would have likely changed the resource levels used in our analyses.

The foster utilization ratio (FUR) is a novel approach to understanding the proportion of dogs living in foster homes relative to all dogs in an organization's care, calculated on a daily level, and allows for utilization analyses across shelters of varying sizes. We anticipated this need as shelters' 2019 canine intake data range from 121 to 6,374 dogs; and during data collection the number of dogs placed in foster care ranged from 13 to 431 (which did not correspond to inventory alone). Despite the strengths of this method, it does not account for changes in intake or outcomes, which could potentially influence FURs, irrespective of increases or declines in foster placements. Nevertheless, FUR is not intended to describe why the proportion of animals living in foster care is changing; but instead to represent on a daily level how the shelter is utilizing its resource of foster homes.

### Conclusion

This study demonstrates that canine foster caregiving increased 1 month into the COVID-19 pandemic as compared to March 2020, but returned to initial levels of foster utilization by June 2020. The available resources of a shelter were related to the types of foster caregivers they relied upon, caregivers with and without prior relationships to the shelter, as well as how shelters primarily placed their dogs: adoption or transferring to other facilities.

New community members fostering for the first time represented the largest proportion of caregivers, and they were much more likely to adopt their fostered dogs than caregivers with a prior relationship to the shelter. Animal welfare organizations would likely save resources and speed the placement of animals into foster homes by regarding new caregivers as individuals fostering with the potential to adopt and reserving training until subsequent foster experiences. Nearly three-quarters of individuals fostering with the intention to adopt adopted their dogs, suggesting that foster-to-adopt and temporary foster programs are beneficial for shelter dog welfare and should be utilized by animal shelters as evidence-based best practices.

Only one quarter of caregivers fostered more than once during the four-month data collection. Over half of foster caregivers were not dog-owning, and resident dogs in the home reduced a caregiver's likelihood of adopting their fostered dog. Behaviorally managed dogs were more frequently returned from foster care for behavioral reasons than dogs without behavioral management, highlighting a need for additional caregiver support for these dogs. Adoption practices, such as foster-facilitated meet-and-greets and adoption from caregiver homes, may reduce dogs' time in foster care, but additional studies are needed to address variables that could not be controlled in this natural experiment.

## Data Availability Statement

Data described in the present study can be found in this online repository: https://dataverse.asu.edu/dataset.xhtml?persistentId=doi:10.48349/ASU/DQLNHM.

## Ethics Statement

The studies involving human participants were reviewed and approved by Arizona State University Institutional Review Board.

## Author Contributions

LG: conceptualized the study and prepared the manuscript. LG, EB, RG, AF, JR, LI, and EF: designed the study methodology. EB, RG, JR, and LI: carried out data collection. EB, RG, JR, LI, and AF: validated data that were collected. LG, RB, and AF: analyzed the data. LG, EB, and RB: visualized the data. LG, EF, and CW: supervised the project and acquired the funding. All authors reviewed and edited the manuscript and have read and agreed to the published version of this manuscript.

## Funding

This study was funded by Maddie's Fund. The funder had no role in data collection, analysis, or interpretation of the results.

## Conflict of Interest

The authors declare that the research was conducted in the absence of any commercial or financial relationships that could be construed as a potential conflict of interest.

## Publisher's Note

All claims expressed in this article are solely those of the authors and do not necessarily represent those of their affiliated organizations, or those of the publisher, the editors and the reviewers. Any product that may be evaluated in this article, or claim that may be made by its manufacturer, is not guaranteed or endorsed by the publisher.
